# Supplementary material for: The thioredoxin system determines CHK1 inhibitor sensitivity via redox-mediated regulation of ribonucleotide reductase activity
Source: Res Sq. 2023 May 2:rs.3.rs-2814118. Preprint. [Version 1] doi: 10.21203/rs.3.rs-2814118/v1 (PMC10187419; doi:10.21203/rs.3.rs-2814118/v1)
Supplement: 1 [file NIHPPrs2814118v1-supplement-1.pdf]

Supplementary Information

Figure S1. Trx1 and TrxR1 is highly expressed in NSCLC tumor samples and is associated with poor prognosis.

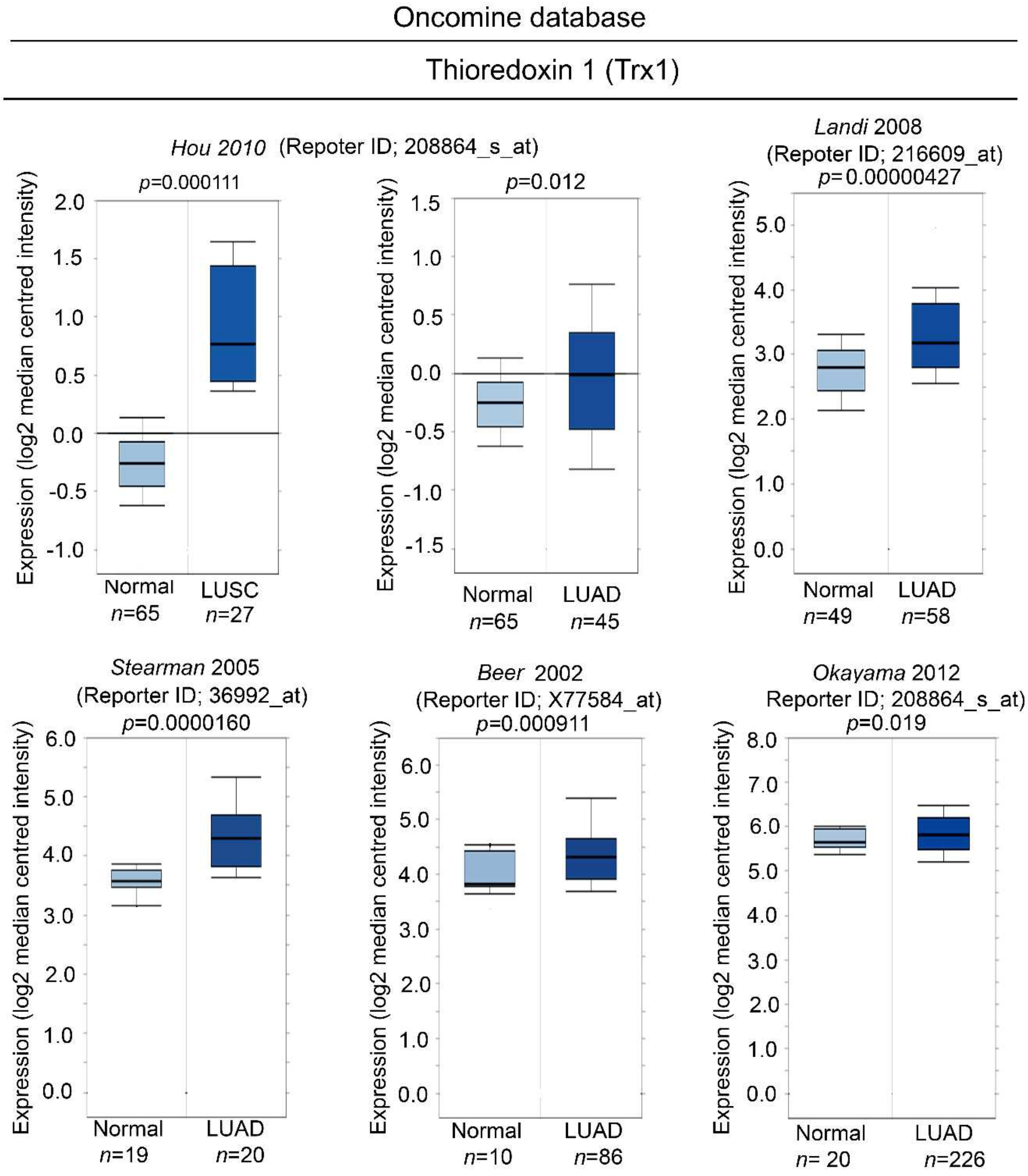

Figure S1A. Expression profile of Trx1 in NSCLC subtypes lung adenocarcinoma (LUAD) and lung squamous cell carcinoma (LUSC) that represent different datasets obtained from the Oncomine database. *P* value was determined by a two-tailed Mann–Whitney test.

Oncomine database

Thioredoxin Reductase 1 (TrxR1)

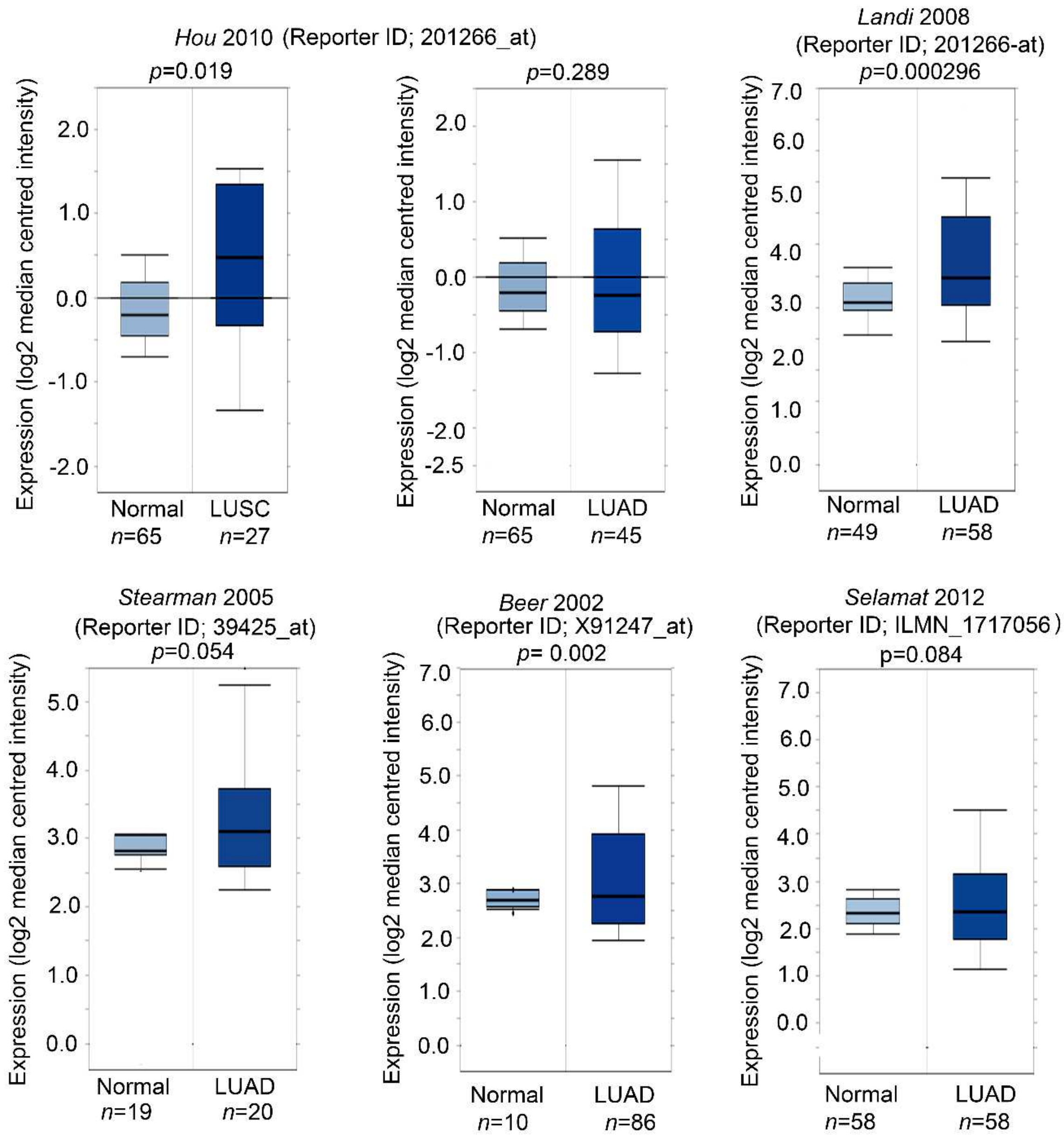

Figure S1B. Expression profile of TrxR1 in LUAD and LUSC that represents different datasets obtained from the Oncomine database. *P* value was determined by a two-tailed Mann–Whitney test.

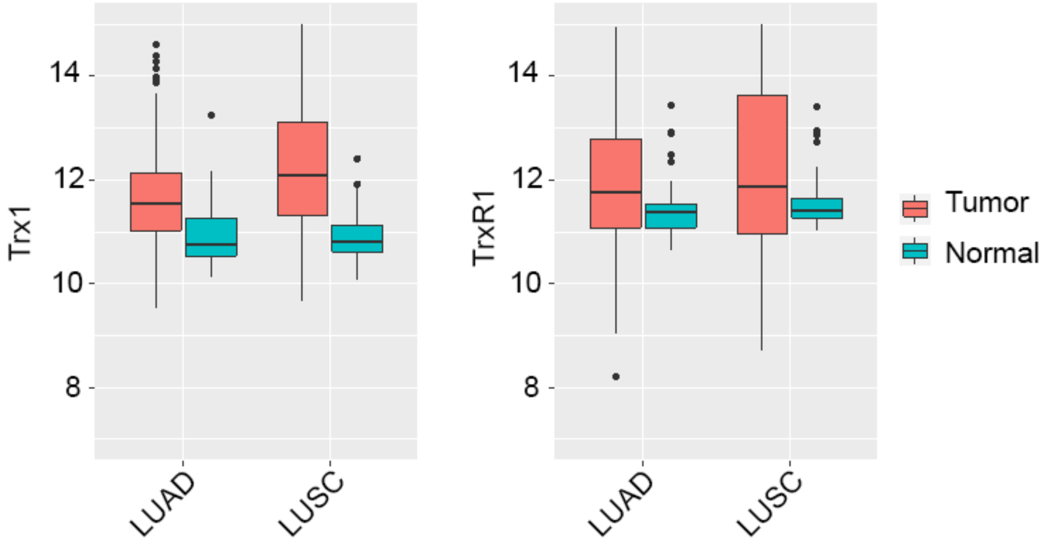

Figure S1C. Expression of Trx1 and TrxR1 in LUAD (N=560; Trx1 p<.00001; TrxR1 p<.00001) and LUSC (N=546; Trx1 <.00001; TrxR1 <.00001), compared to normal tissue. The expression of both Trx1 and TrxR1 in more than 50% of NSCLC patients are higher than the 75% quantile expression level the normal samples. The data is from TCGA.

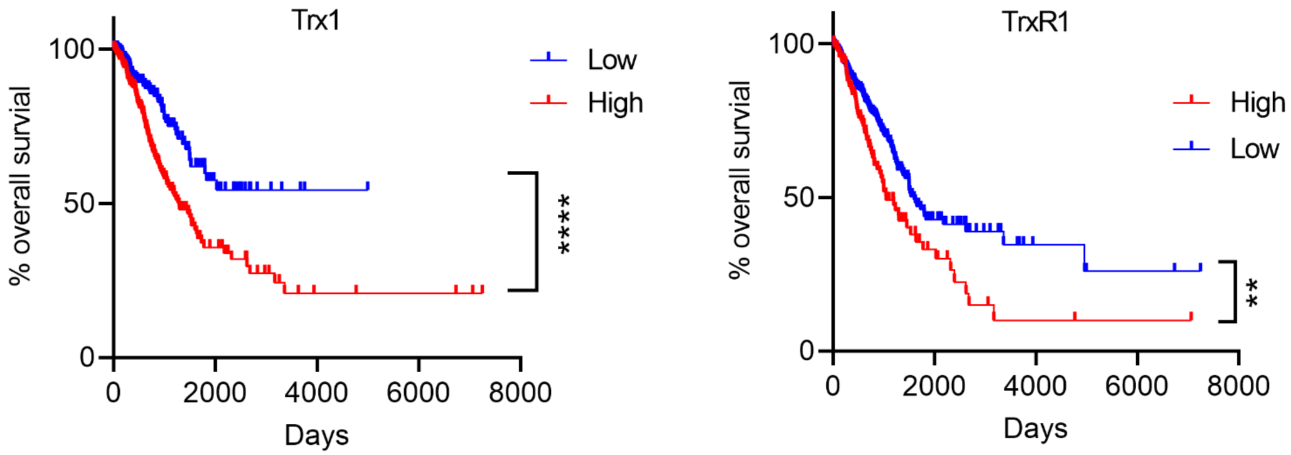

Figure S1D. Survival curves (Kaplan-Meier) and the association of higher Trx1 and TrxR1 expression with poor overall survival in patients with LUAD (Trx1; **** $p < 0.0001$; TrxR1; ** $p = 0.0017$). The data is from TCGA.

Figure S2.

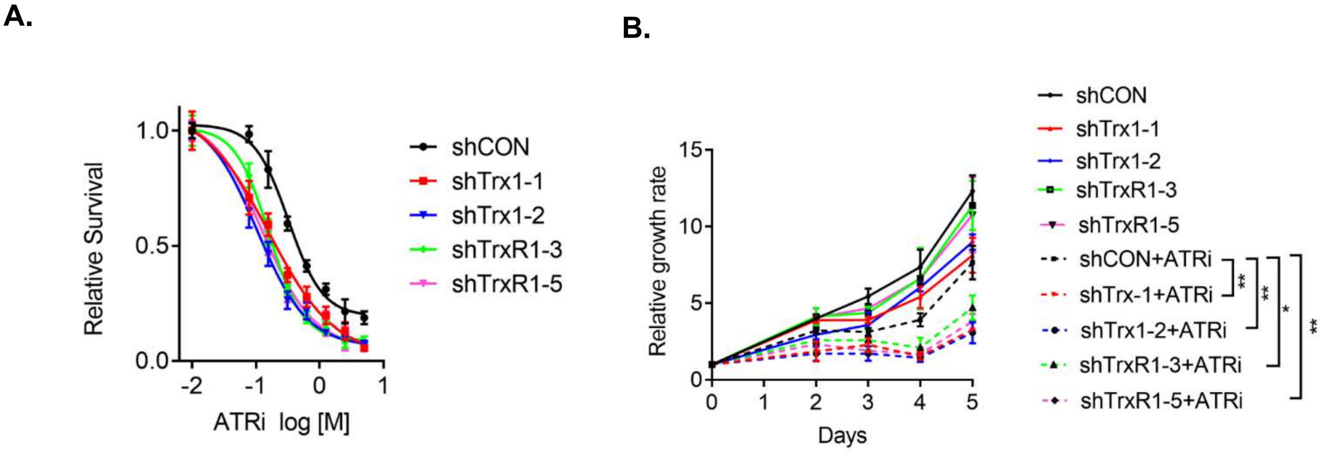

Figure S2. Trx1 or TrxR1 depletion increases the sensitivity of an ATR inhibitor. **(A)** Cell growth after dose-dependent treatment with ATRi (VE821) of H1299 cells knocked down for Trx1 or TrxR1 expression. **(B)** The growth rate of H1299 cells treated with an ATRi (1μM) for 24 h and depleted for Trx1 or TrxR1 expression. [Statistical information: n=3, All histograms depict mean value; error bars represent ± SD. The *p- value*s were calculated using one way ANOVA for multiple comparison; **p*≤0.05; ** *p≤0.005*].

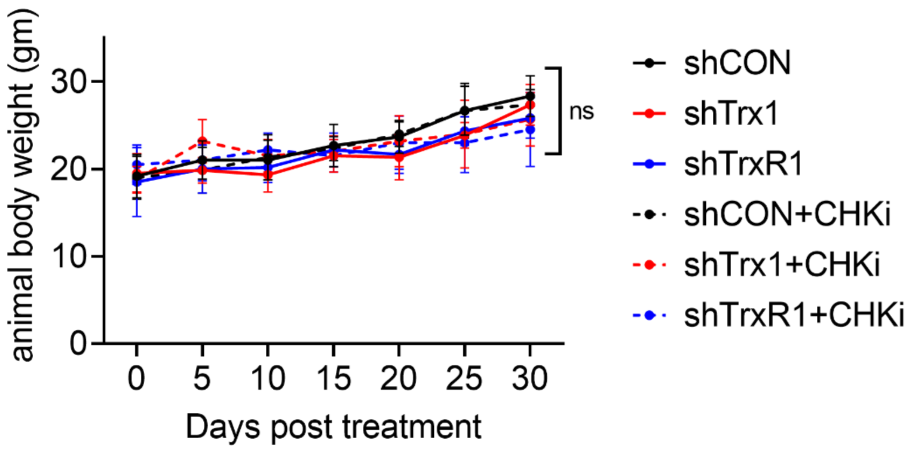

Figure S3. Mouse body weight is similar among the experimental groups from Figure 2, E, F, G. Effect of CHK1i treatment on overall body weight of animals in the indicated groups. [Statistical information: n=3, All histograms depict mean value; error bars represent ± SD. The *p-value*s were calculated using one way ANOVA for multiple comparison; *ns: non-significant*]

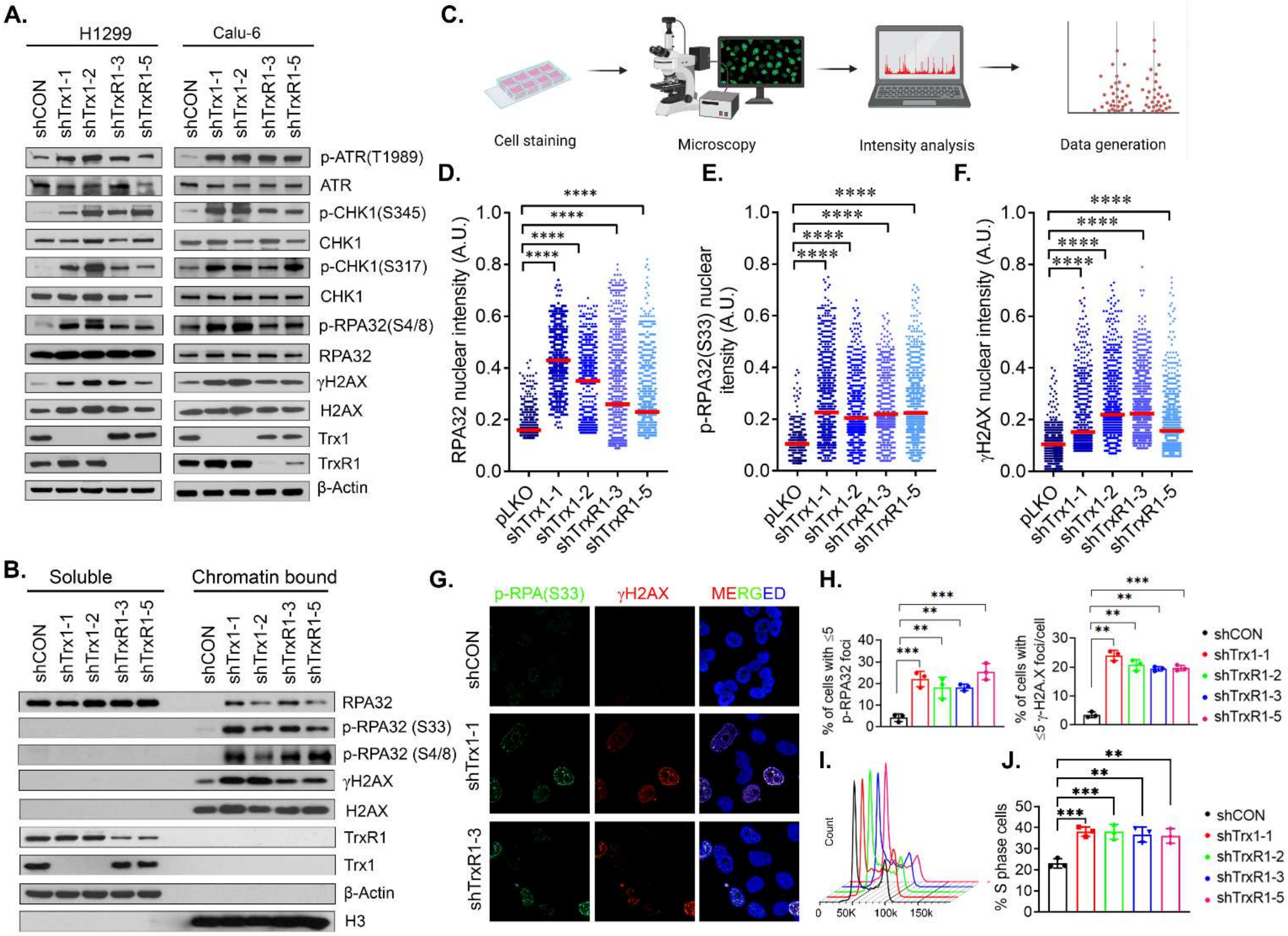

A.
H1299
Calu-6
shCON
shTrx1-1
shTrx1-2
shTrxR1-3
shTrxR1-5
p-ATR(T1989)
ATR
p-CHK1(S345)
CHK1
p-CHK1(S317)
CHK1
p-RPA32(S4/8)
RPA32
γH2AX
H2AX
Trx1
TrxR1
β-Actin
B.
Soluble
Chromatin bound
RPA32
p-RPA32 (S33)
p-RPA32 (S4/8)
γH2AX
H2AX
TrxR1
Trx1
β-Actin
H3
C.
Cell staining
Microscopy
Intensity analysis
Data generation
D.
RPA32 nuclear intensity (A.U.)
E.
p-RPA32(S33) nuclear itensity (A.U.)
F.
γH2AX nuclear intensity (A.U.)
pLKO
G.
p-RPA(S33)
γH2AX
MERGED
H.
% of cells with ≤5 p-RPA32 foci
% of cells with ≤5 γ-H2A.X foci/cell
I.
Count
0
50K
100k
150k
J.
% S phase cells

K.

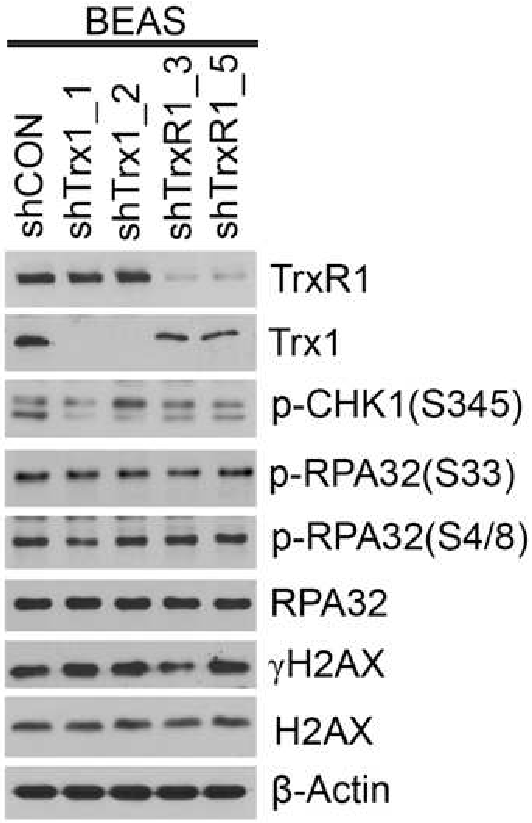

Figure S3. Trx1 or TrxR1 depletion increases replication stress (RS).

(**A**). Representative western blots of RS marker proteins in H1299 and Calu-6 cells depleted for Trx1 or TrxR1.

(**B**) Representative western blots of the DNA-binding protein RPA32 and phosphorylated RPA32(p-RPA32 S33 and S4/8) and γH2AX in H1299 cells with Trx1 or TrxR1 depletion. Cytoplasmic and chromatin fractions were fractionated using mild and stringent detergent-based buffers respectively.

(**C**) An illustration of the scheme to measure nuclear intensity of RS markers.

(**D-F**) The nuclear intensity of RPA32, p-RPA32(S33) and γH2AX in H1299 cells **[AU: which cells?]** with Trx1 or TrxR1 depletion.

(**G and H**) Representative immunofluorescence images of p-RPA32(S33) and γH2AX staining in the indicated groups. Bar graphs shows the frequency of cells with ≥5 foci/cell in the indicated groups.

(**I and J**) The degree of accumulation of cells in the S phase upon Trx1 or TrxR1 depletion by flow cytometric profile of cell cycle progression in the indicated groups (I) and its quantitation (J).

(**K**) Representative western blots of RS-related proteins in untransformed human normal epithelia BEAS cells upon Trx1 or TrxR1 depletion.

[Statistical information: n=3, All histograms depict mean value; error bars represent ± SD. The *p-values* were calculated using one way ANOVA for multiple comparison. Red line in dot plot indicates mean; ** *p≤0.005; *** p ≤0.001; **** p ≤0.0001*].

Figure S4.

A.

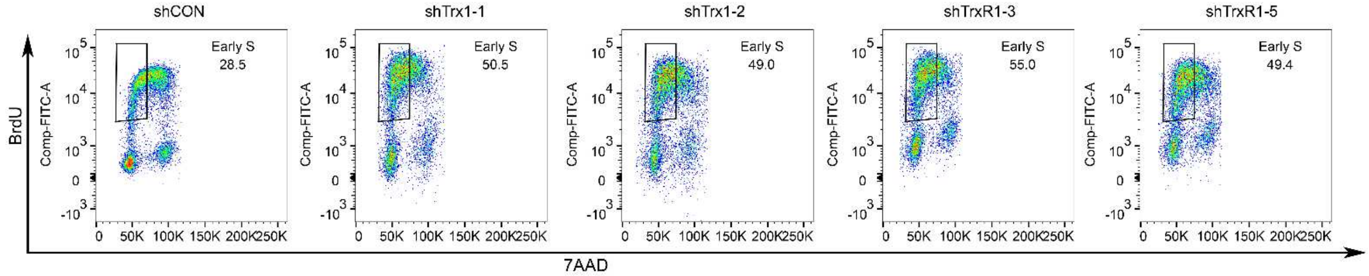

B.

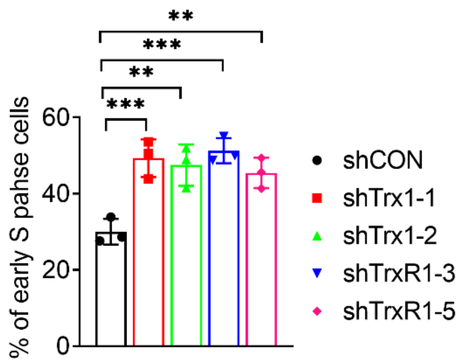

Figure S4. Trx1 or TrxR1 depletion leads to the accumulation of cells in the early S phase.

(**A**). Flow cytometric dot plots to measure BrdU uptake in actively proliferating cells in the indicated groups.

(**B**) Percent of cells in early S phase upon Trx1 or TrxR1 depletion. Bar graph shows the quantification of flow cytometric data from Figure S4A.

[Statistical information: n=3, All histograms depict mean value; error bars represent ± SD. The *p-value*s were calculated using one way ANOVA for multiple comparison. Red line in dot plot indicates mean; ** *p≤0.005;* *** *p ≤0.001*].

Figure S5.

A.

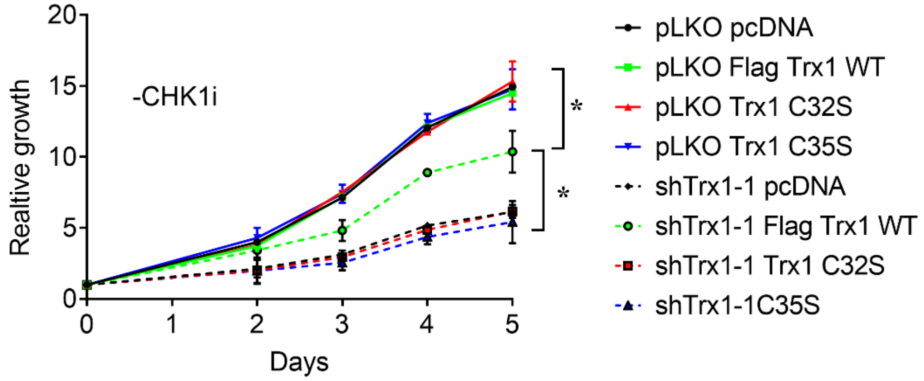

B.

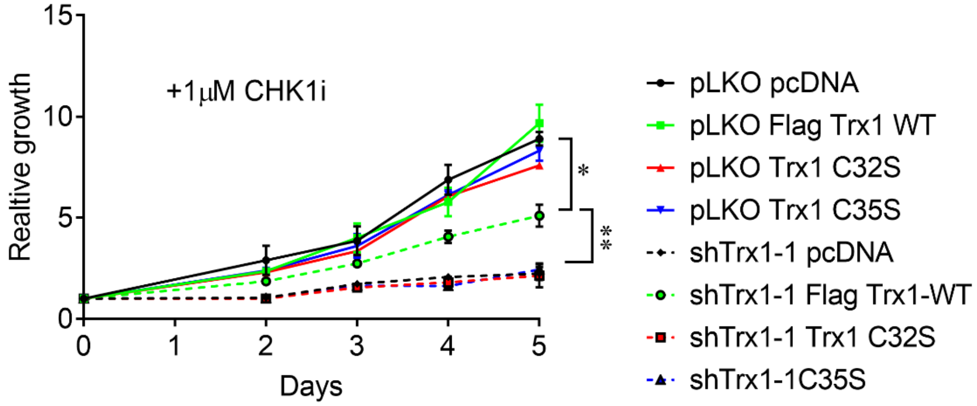

Figure S5. RRM1-WT, but not RRM1 redox mutant expression, partially abrogates Trx1 depletion-induced slow cell proliferation and the sensitivity to CHKi.

(**A**) Relative growth of cells expressing Trx1-WT or its redox mutants after Trx1 depletion.

(**B**) Relative growth of cells expressing Trx1-WT or its redox mutants in the presence of a CHK1i inhibitor with or without Trx1 depletion.

[Statistical information: n=3, All histograms depict mean value; error bars represent ± SD. The *p-values* were calculated using one way ANOVA for multiple comparison; *$p \leq 0.05$; ** $p \leq 0.005$]

Figure S6.

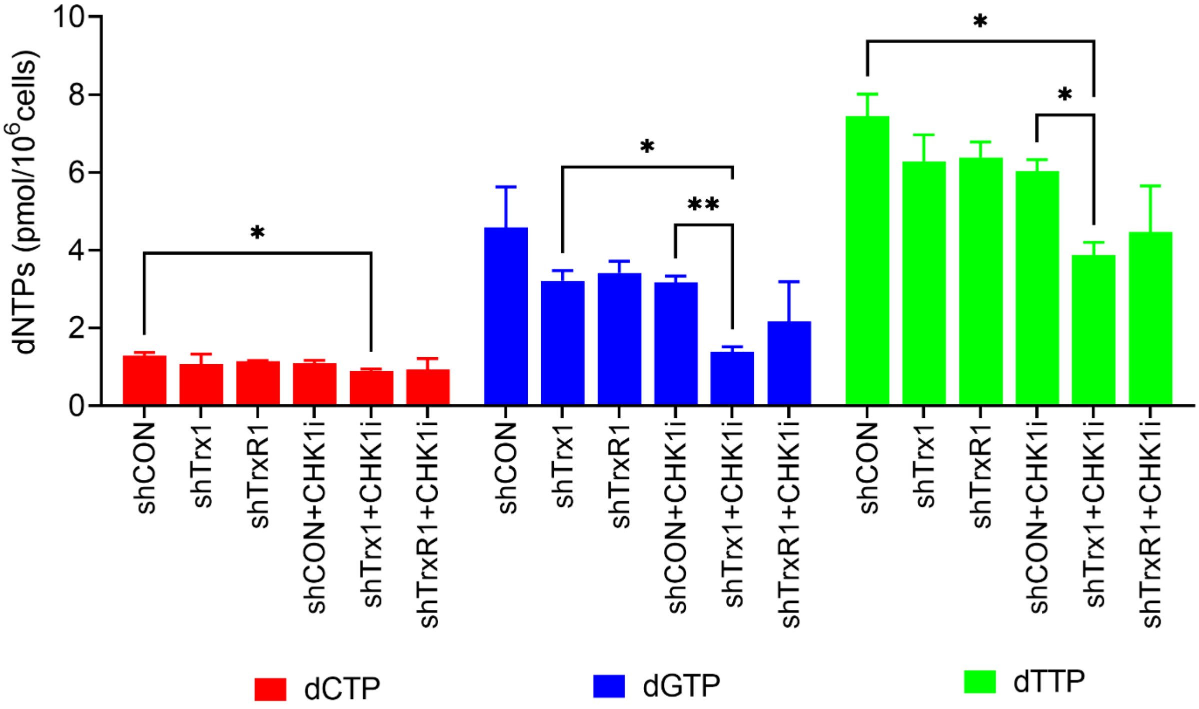

Figure S6. Levels of the indicated dNTPs in cells with Trx1 or TrxR1 depletion with or without in combination of CHK1 inhibition. Total cellular dNTPs were subjected to RT-based primer extension assay to quantify each dCTP, dGTP and dTTP concentration.

[Statistical information: n=3 All histograms depict mean value; error bars represent ± SD. The *p-values* were calculated using one way ANOVA for multiple comparison; *$p \leq 0.05$; ** $p \leq 0.005$]

Figure S7.

A.

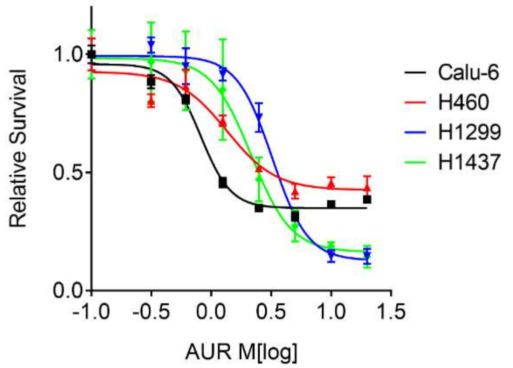
Relative Survival
1.0
0.5
0.0
-1.0
-0.5
0.0
0.5
1.0
1.5
AUR M[log]
Calu-6
H460
H1299
H1437

B.

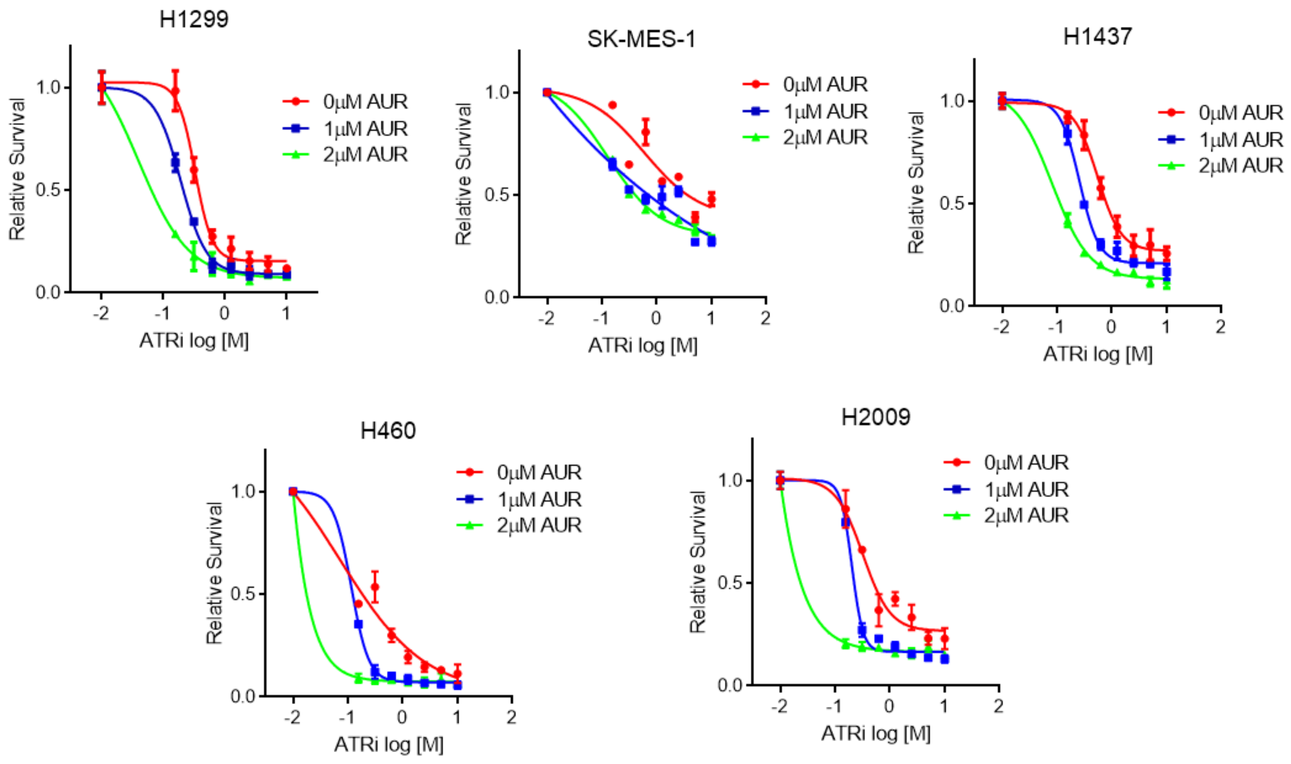
H1299
SK-MES-1
H1437
H460
H2009
Relative Survival
ATRi log [M]
0μM AUR
1μM AUR
2μM AUR

C.

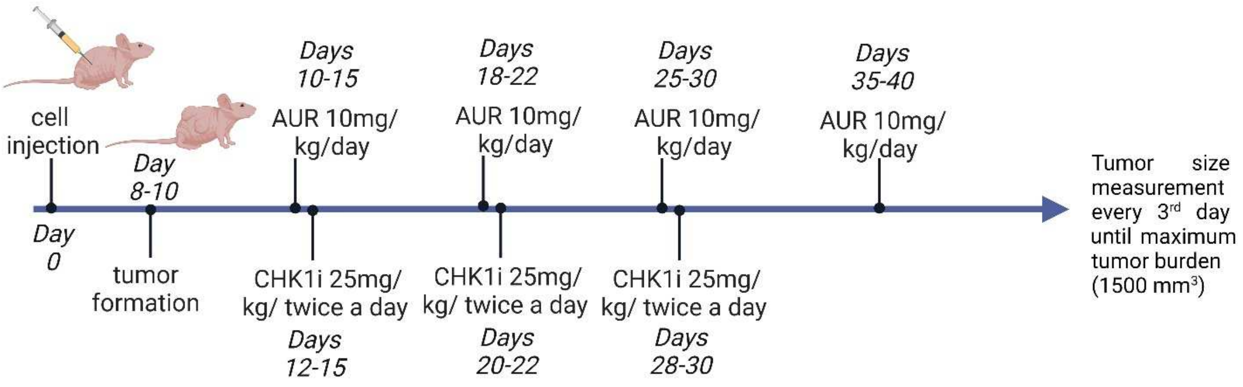

Figure 7S. The synergistic interaction of AUR and CHK1i or ATRi.

(**A**) The relative survival of different NSCLC cell lines treated with AUR in a dose dependent manner.

(**B**) The relative survival of different NSCLC cell lines treated with ATRi and AUR.

(**C**) Graphical representation of the scheme of drug administration in the xenograft model.

Figure S8

A.

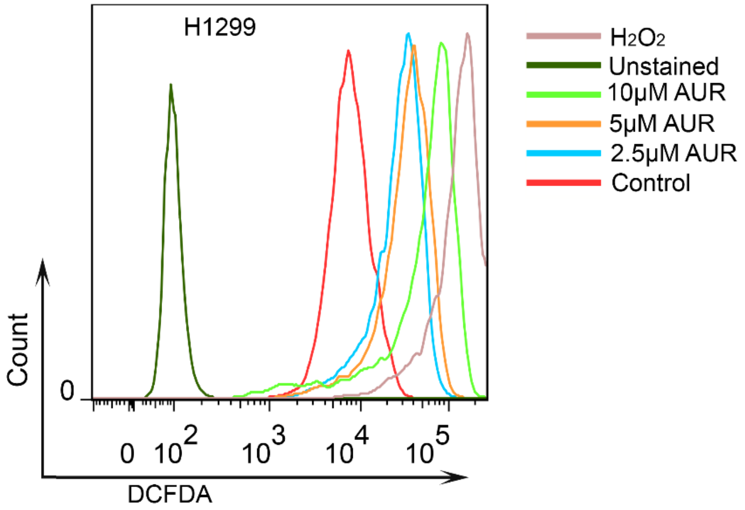

B.

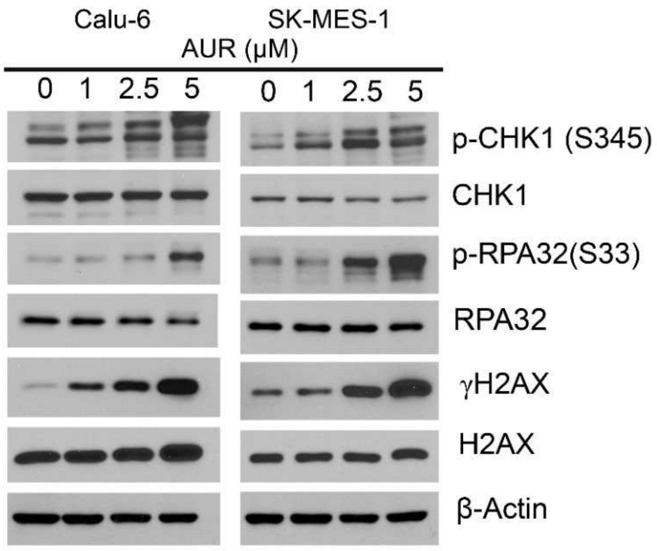

Figure S8. AUR treatment leads to an increased intracellular level of ROS and increased RS.

(**A**) Flow cytometric profile of DCFDA intensity in the indicated groups. H_2O_2 was used as a control.

(**B**) Representative western blots of RS marker proteins in the indicated NSCLC cell lines treated with AUR.
